# Supplementary material for: The causal relationship between the human gut microbiota and pyogenic arthritis: a Mendelian randomization study
Source: Front Cell Infect Microbiol. 2024 Nov 26;14:1452480. doi: 10.3389/fcimb.2024.1452480 (PMC11629706; doi:10.3389/fcimb.2024.1452480)
Supplement: Supplementary file 1 [file DataSheet1.zip › mendelian test/ebi-a-GCST90027550.csv_leave_one_out.pdf]

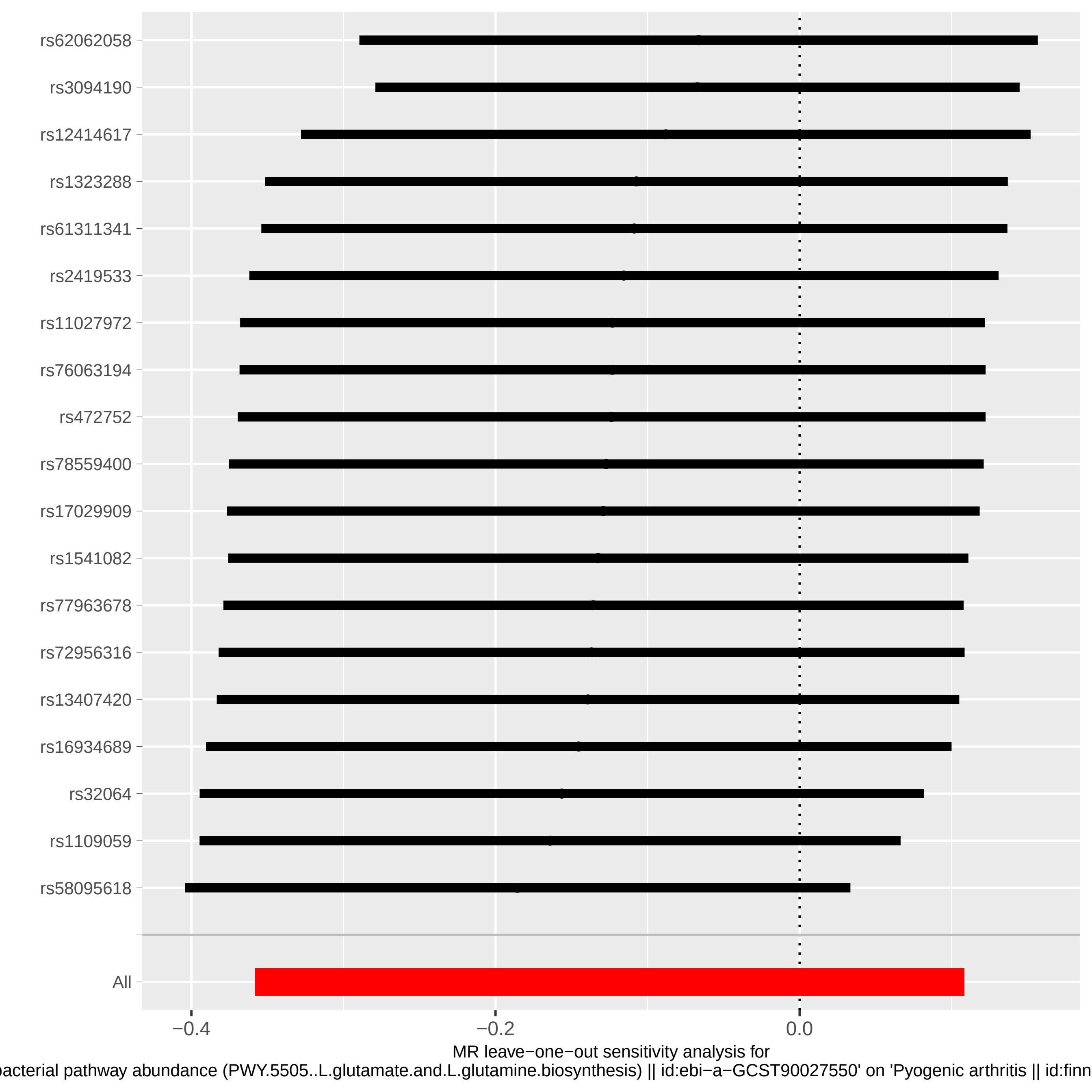
rs62062058
rs3094190
rs12414617
rs1323288
rs61311341
rs2419533
rs11027972
rs76063194
rs472752
rs78559400
rs17029909
rs1541082
rs77963678
rs72956316
rs13407420
rs16934689
rs32064
rs1109059
rs58095618
All
−0.4
−0.2
0.0
MR leave−one−out sensitivity analysis for
bacterial pathway abundance (PWY.5505..L.glutamate.and.L.glutamine.biosynthesis) || id:ebi−a−GCST90027550' on 'Pyogenic arthritis || id:finn
